# Prospective evaluation of intimate partner violence in fracture clinics (PRAISE-2): protocol for a multicentre pilot prospective cohort study

**DOI:** 10.1186/s40814-018-0301-9

**Published:** 2018-06-15

**Authors:** Kim Madden, PRAISE-2 Investigators, Sheila Sprague, Brad A. Petrisor, Taryn Scott, Diane Heels-Ansdell, Michelle Ghert, Lehana Thabane, Mohit Bhandari, Herman Johal, Bill Ristevski, Matthew Denkers, Brian Drew, Kerry Tai, Priyancee Sabhaya, Caley Laxer, Cody Kjell, Tristiana Dalchand, Gina Del Fabbro, Jeremy A. Hall, Aaron Nauth, Michael McKee, Daniel Whelan, Sarah Ward, Amit Atrey, Milena ( Lynn) Vicente, Jennifer Hidy, Paril Suthar, Melanie MacNevin, Prism Schneider, Ryan C. Martin, Richard Buckley, Robert Korley, Paul Duffy, Leah Schultz, Tanja Harrison, Aftab Akbari, Ydo V. Kleinlugtenbelt, Elvira R. Flikweert, Ellie B. M. Landman, Cherelle V. van Stenus, Annemarie S. E. Brandsma, Maria Villar Caesares, Ernesto Guerra Farfán, Vicente Molero García, Jordi Teixidor Serra, Jordi Tomas Hernández, Jordi Selga Marsà, Juan Antonio Porcel Vazquez, Jose Vicente Andres Peiró, Jaume Mestre Torres, Pilar Lalueza Broto, Yaiza García Sanchez, Andrea Perales García, Felipe Moreira Borim, Míriam Garrido Clua, Elisa A. M. Hackenberg, Virve Koljonen, Teppo Järvinen

**Affiliations:** Department of Health Research Methods, Evidence, and Impact, McMaster University, 293 Wellington St N Suite 110, Hamilton, ON L8L 8E7 Canada

**Keywords:** Intimate partner violence, Domestic violence, Orthopaedic surgery, Cohort studies, Protocol, Feasibility

## Abstract

**Background:**

One third of women experience intimate partner violence (IPV) in their lifetime. Orthopaedic health care professionals are in a good position to identify women experiencing escalating physical violence and act to promote their immediate safety, connect them to IPV resources, and reduce the risk of further harm. However, there have been no studies that explore whether experiencing a musculoskeletal injury can trigger or worsen IPV, and there have been no studies on how experiences of IPV affect orthopaedic outcomes. The primary objective of the PRAISE-2 pilot study is to assess the feasibility of conducting a large cohort study to determine the association between IPV and injury-related complications. The secondary clinical objectives are to preliminarily explore how a history of IPV affects orthopaedic outcomes and how patterns of IPV change over time following an orthopaedic injury.

**Methods:**

We will complete a pilot multicentre prospective cohort study of 250 women with musculoskeletal injuries to determine the feasibility of a multinational prospective cohort study that will determine if prior or ongoing IPV affects orthopaedic outcomes following an injury, and how patterns of IPV change over time. Our primary outcome is feasibility measured using recruitment rate (success criterion 50 patients/site in 12 months), adherence to visit windows (success criterion 75%), participant retention (success criterion 85%), and data completeness (success criterion 80%). Our secondary exploratory clinical outcomes are injury-related complications, return to function, new IPV disclosures, utilization and cost of support services, changes in abuse patterns, quality of life, and readiness to make relationship changes. We will assess feasibility based on pre-defined criteria for feasibility success and we will analyze secondary outcomes in an exploratory fashion.

**Discussion:**

The PRAISE-2 pilot study is the first step toward determining how experiences of IPV affect orthopaedic outcomes such as injury-related complications. This study will determine feasibility and assist in the development of large-scale multinational prospective IPV studies for our future IPV research program. This study will engage health care professionals from around the world to increase awareness of how IPV affects patients’ musculoskeletal and injury outcomes.

**Trial registration:**

clinicaltrials.gov NCT02529267. Registered 20 Aug 2015.

## Background

Intimate partner violence (IPV) is a pattern of physical, emotional, or sexual abuse or controlling behaviours committed by a current or past spouse or romantic/sexual partner [1]. The World Health Organization reports that one in three women globally will experience physical or sexual IPV or domestic abuse in their lives [1]. Every 6 days, a woman in Canada is killed by her intimate partner [2]. IPV is a prevalent social issue which poses significant health concerns. IPV disproportionately affects women and is a leading cause of non-fatal injury in females in North America [3, 4]. The cost of IPV in Canada is estimated at $7.5 billion annually [5]. IPV victims experience more physical and mental health problems [6, 7], including musculoskeletal injuries [8, 9], and use health care resources more frequently than non-abused women [10, 11]. A recent systematic review of 37 IPV prevalence studies reported that nearly one in four women presenting to emergency medicine and family medicine experienced IPV in their lifetime [12].

Recently, attention has started to focus on fracture clinics as an environment in which IPV is important to patient care. Health care professionals in fracture clinics have an important opportunity to identify and assist women experiencing IPV as they are well-positioned to discuss IPV with women who have presented with an injury. Compared to emergency physicians who tend to see patients once for an injury, orthopaedic surgeons often develop long-term interactions with patients over repeated follow-up clinic visits which serves to foster trust and disclosure [13]. The original PRAISE study [14], which included 2945 women globally, found that one in six women in fracture clinics experienced IPV in the year prior to completing the study. Additionally, we found that one in three injured women have experienced IPV in her lifetime [14]. Our previous research has found that IPV is more prevalent in fracture clinics within Ontario Level I Trauma Centres than in many other medical specialties [15] and the lifetime prevalence globally is similar in fracture clinics, emergency medicine, and family medicine [12, 14]. Major orthopaedic associations, such as the Canadian Orthopaedic Association [13] and American Academy of Orthopaedic Surgeons [16], have created position statements on care of patients who have experienced IPV, and they are advocating strongly for increased awareness of IPV among health care professionals who care for women with injuries.

Orthopaedic health care professionals are in a good position to identify women experiencing escalating physical violence (with resultant musculoskeletal injuries) and act to promote their immediate safety, connect them to IPV resources, and reduce the risk of further harm. It has been hypothesized that the severity of physical abuse among women presenting to fracture clinics may be higher than in other specialties [14]. Escalation of physical violence remains a key risk factor for intimate partner homicide [17]. More than one third of female homicides globally are perpetrated by an intimate partner [18] and 45% of women who are killed by their intimate partner present to a hospital for treatment of IPV-related injuries in the 2 years before their death [19]. Based on the above evidence, we argue that fracture clinics are instrumental to identifying women with more severe cases of IPV who are at greater risk of severe injury and homicide. However, more information is needed on how experiencing IPV affects musculoskeletal outcomes.

Although there is a large body of research on mental health [20] and reproductive health [21] outcomes following IPV, Sanchez-Lorente and colleagues [22] note that there is very little information on how IPV experiences affect specific physical health outcomes. Most existing studies on IPV and physical health, including the original PRAISE study, are either cross-sectional (therefore cannot assess longer-term outcomes) or report on general self-reported psychosomatic outcomes like gastrointestinal distress and headaches [22]. The fields of mental health and reproductive/maternal health have high-quality data on specific objective outcomes such as low birth weight, miscarriage, and HIV/AIDS infection [23, 24]. There is a need for studies that focus on the specifics of how IPV experiences affect physical health and objective outcomes among injured women, such as injury-related complications. If health care professionals have specific information about how IPV affects injury-related outcomes, they will be more likely to consider how IPV is affecting their patients and may be in a position to offer more individualized care. Evidence that IPV directly affects patient outcomes is necessary in order to change practice patterns of orthopaedic surgeons and other health care professionals who treat injured women. For example, health care professionals routinely inquire about smoking history when they are evaluating injured patients as there is strong evidence that smoking is associated with poor fracture healing [25]. If we have similar direct evidence that IPV affects patients’ injury outcomes, health care professionals who treat women with injuries may be more inclined to inquire about IPV routinely and potentially to adjust their treatment plan accordingly.

### Rationale for a pilot study

We chose to conduct a pilot study to assess the feasibility of enrolling and following patients in our target population and to assess the feasibility of our study procedures. Lessons learned from this pilot study will inform future prospective studies in our IPV research program. While our group was successful in recruiting patients for the original PRAISE study, that study was cross-sectional. We believe that it may be more difficult to enroll patients in a longitudinal IPV study because there is less anonymity compared to a cross-sectional study. There are also additional ethical and safety issues to consider with longitudinal IPV research compared to anonymous cross-sectional IPV studies which may affect follow-up rates. Therefore, this pilot study is needed to test the feasibility of our procedures to maximize follow-up rates while maintaining participant safety. Additionally, this pilot study will be able to inform future longitudinal IPV research on which outcomes are feasible to measure over a 1-year period in this population.

### Research objectives

#### Feasibility objectives

The primary objective of the PRAISE-2 pilot study is to determine the feasibility of a multinational prospective cohort study. Specifically, we will (1) assess our ability to recruit women across clinical sites; (2) evaluate adherence to study visit windows; (3) assess our ability to follow participants and collect data for 12 months; (4) assess our ability to collect data on our chosen clinical outcomes, including questionnaire completion; and (5) identify areas for improvement for future studies.

#### Clinical objectives

Clinical objectives in this pilot study are exploratory. The clinical objectives of the PRAISE-2 study include determining (1) how a history of IPV affects injury-related complications; (2) how a history of IPV affects return to pre-injury function; (3) incident cases of IPV after a musculoskeletal injury if the injury was not the result of IPV; (4) how a history of IPV affects health care and support service use after a musculoskeletal injury; (5) how a history of IPV affects health-related quality of life after a musculoskeletal injury; (6) how patterns of IPV change over time after a musculoskeletal injury; and (7) how abused women’s stage of change (i.e. readiness to make changes to move toward a life free from violence) changes over time after a musculoskeletal injury.

## Methods

This study was registered with clinicaltrials.gov (NCT02529267) on 20 August 2015, before the first participant was enrolled.

### Overview of the design

We plan to complete a multicentre pilot prospective cohort study of 250 women with musculoskeletal injuries to assess our ability to recruit and follow our target population, and to measure our clinical outcomes.

Important design and organizational aspects of the proposed PRAISE-2 study include (1) leveraging the international interest in IPV in surgical settings from the original PRAISE study to strengthen buy-in for this pilot cohort study; (2) broad eligibility criteria (all adult females with musculoskeletal injuries who are able to provide informed consent in a private location) ensures wide applicability of our findings beyond the centres involved; and (3) a comprehensive plan to maximize follow-up rates while maintaining patient safety.

### Patient selection

#### Eligibility criteria

We will use broad eligibility criteria to increase the generalizability of the study. The inclusion criteria are (1) adult females (at least 16 or 18 years of age depending on local ethics requirements); (2) patients presenting to participating fracture clinics within 6 weeks of their injury; and (3) patients presenting with a fracture or dislocation which is being managed with either surgical or non-surgical treatment.

The exclusion criteria are (1) unwilling to or unable to provide consent; (2) unable to complete the study questionnaires in a private location, due to safety and confidentiality; (3) unwilling or unable to follow the study protocol or their attending surgeon has concerns about their ability or willingness to follow study protocols; and (4) does not speak and write in English or the dominant language of the local clinic. Due to the sensitive nature of the topic, only patients who can consent for themselves will be considered for participation.

#### Patient screening and enrolment

All new female patients (within 6 weeks of injury) presenting to the fracture clinics of participating surgeons will be screened for participation in this study. A female research coordinator will approach each potentially eligible female patient and screen each patient for eligibility. The female research coordinator will obtain informed consent from each eligible patient who wishes to participate. Since this study will record change in IPV status over time, and we plan to make comparisons to a non-abused control group, we will follow all eligible and consenting patients regardless of whether they report experiencing IPV at baseline. We will record numbers of excluded and missed patients, and reasons for exclusion.

### Study outcomes

#### Primary (feasibility) outcomes

The primary outcome of the pilot study is feasibility including recruitment, adherence to visit windows, participant retention, and data completeness. The criteria for success of feasibility are as follows:

- Recruitment—Our recruitment strategy will be considered feasible if each site is able to recruit 50 participants in 12 months or less after their training call/visit.
- Adherence to visit windows—We have attempted to align study visit windows with clinical standard of care visits; however, standard of care visits can vary by region and/or type of injury. We expect that at least 75% of study visits should be within the defined windows in Table 1.
- Participant retention—While 20% loss to follow-up has traditionally been considered the industry standard [26], there is evidence from orthopaedic studies that bias begins to affect study results at even lower rates of loss to follow-up [27]. Therefore, our loss to follow-up minimization strategy will be considered feasible if loss to follow-up remains under 15%.
- Data completeness—Based on our experience in previous multicentre orthopaedic trauma studies, we are typically able to obtain questionnaire completeness rates of 75–85% at 12 months. Therefore, we will consider our data collection strategies to be feasible if questionnaire completion remains over 80%.

**Table 1:** PRAISE-2 schedule of events

Assessment	Baseline (0–6 weeks from injury)	1 month (2–6 weeks from injury)	3 months (11–15 weeks from injury)	6 months (24–28 weeks from injury)	12 months (48–56 weeks from injury)
Target visit window					
Screening form	X				
Informed consent	X				
Demographic characteristics form	X				
Injury characteristics form	X				
IPV status (type, frequency, severity)	X	X	X	X	X
Assessment for complications	X	X	X	X	X
Support service utilization	X	X	X	X	X
Return to function	X	X	X	X	X
EQ-5D	X	X	X	X	X
Stages of change	X	X	X	X	X
Radiograph	(X)	(X)	(X)	(X)	(X)
Clinic notes	(X)	(X)	(X)	(X)	(X)

(X) = Only if required to adjudicate an adverse event

#### Secondary (clinical) outcomes

Since this is a pilot study, clinical outcomes will be exploratory. Clinical outcomes are (1) injury-related complications; (2) return to pre-injury function; (3) new IPV disclosures; (4) utilization and associated costs of health, legal, and social support services; and (5) changes in abuse severity/frequency and type of abuse (physical, emotional, and/or sexual IPV); (6) health-related quality of life; and (7) stage of change.

- Injury-related complications—Injury-related complications are adverse effects that are related to sustaining the injury or the treatment for the injury. These include bone healing problems, infection, unplanned additional surgical procedures or hospitalizations, mortality, and problems with the surgical implants requiring medical intervention. An orthopaedic surgeon who is blinded to identifying details and IPV status will adjudicate whether the event is injury-related.
- Return to pre-injury function—We will use the Return to Function Questionnaire (RTF) which is a four-question tool that was used in a recently completed large FDA-regulated fracture trial [28].
- New IPV disclosures—Women’s self-reported experience of IPV will be measured using a direct method of screening used by the PRAISE Investigators [14, 15] in two previous studies conducted in trauma populations. The tool comprises three questions with three response options (Table 2). This tool has proven feasible to administer in a trauma population and has been shown to have greater sensitivity to identify IPV compared to the partner violence screen (PVS) [29]. It is important to maximize sensitivity when screening IPV victims because not identifying victims can have many negative health and social consequences [30]. The direct method of screening can also distinguish between types of IPV (i.e. physical, sexual, and emotional abuse). A participant will be considered to have disclosed IPV if she answers positively to at least one of the three direct screening questions.
- Use and associated costs of health, legal, and social support services—Women’s access to and use of health and support services will be measured by directly asking participants to self-report if they have accessed health care services, a social worker, mental health professional, women’s shelter, helpline, violence against women website, or legal assistance. We will further ask participants whether they have referred a friend or family member to any of the IPV services. We will conduct a cost analysis from a societal perspective using a 1-year time horizon. Total and incremental costs will be reported as 2018 Canadian dollars for women with and without a history of IPV. Direct costs will be estimated from costs associated with injury-related complications and additionally derived from utilization of health care, social work, and mental health services. Indirect costs will be estimated from loss of productivity and time to return to function.
- Changes in abuse type and severity/frequency—Using the direct method of screening (Table 2), which categorizes types of violence as physical, emotional, and/or sexual abuse, we will record and analyze changes in type and severity/frequency of IPV experienced over time. Participants will be classified as experiencing no abuse, stable level of abuse, escalating abuse, and de-escalating abuse.
- Health-related quality of life—Participants’ quality of life will be measured using the EuroQol-5 Dimensions (EQ-5D), a widely used and well-validated quality of life tool [31]. The EQ-5D is a comprehensive, five-item compact health status classification and health state preference questionnaire.
- Stage of change—Participants will complete the Domestic Violence Survivor Assessment (DVSA) Short Form questionnaire to determine her stage of change. The stages of change are based on the transtheoretical model of health behaviour change applied specifically to survivors of abuse [32]. The stages of change are (1) Pre-contemplation: committed to continuing the relationship, change is not contemplated; (2) Contemplation: committed but questioning/contemplating change; (3) Preparation: considering change/exploring options to end abuse; (4) Action: victim breaks away from abusive relationship or partner stops being abusive, and (5) Maintenance: establishment of a new life apart or together. The original DVSA is well-used in IPV research and counselling and has been determined to be feasible to administer, reliable, and sensitive to change [33]. We developed the DVSA Short Form for the purpose of this study, with the aim of making the self-administered form more accessible and less time-consuming to administer in a research setting (e.g. removed gendered pronouns, removed references to having children, simplified language, removed two redundant/obsolete questions). Depending on the findings regarding feasibility of administration, we may conduct validation studies in the future.

**Table 2:** Questions on the direct method of IPV screening questionnaire

Question	Response options		
*In the past year…*			
Have you been physically abused by your intimate partner?	Often	Sometimes	Never
Have you been emotionally abused by your intimate partner?	Often	Sometimes	Never
Have you been sexually abused by your intimate partner?	Often	Sometimes	Never
*In your lifetime…*			
Have you been physically abused by your intimate partner?	Often	Sometimes	Never
Have you been emotionally abused by your intimate partner?	Often	Sometimes	Never
Have you been sexually abused by your intimate partner?	Often	Sometimes	Never

### Study follow-up

**Fig. 1:**
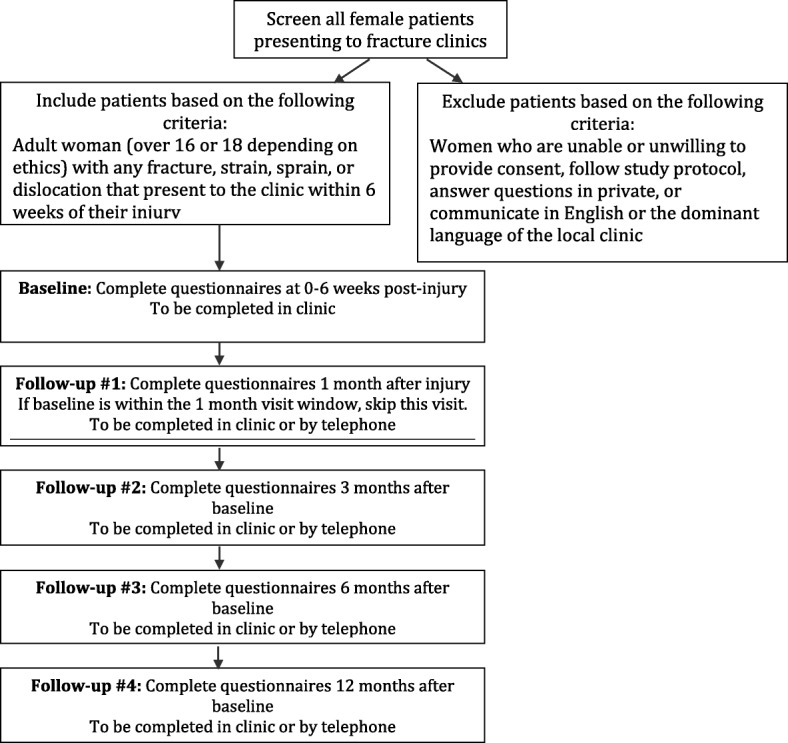
PRAISE-2 study process overview

Participants will complete the questionnaires in the surgical clinic (baseline), and at 1, 3, 6, and 12 months after the baseline assessment. Participants will be provided with the option of completing the study questionnaire in the clinic or over the telephone (Fig. 1). Table 1 lists the assessments at each study time point. We chose a 12-month follow-up period because this is the most common follow-up length in other IPV studies [34].

### Study location and clinical sites

The PRAISE-2 Methods Centre will be located at McMaster University’s Centre for Evidence-Based Orthopaedics. The Centre for Evidence-Based Orthopaedics is a well-established research group specializing in large-scale multicentre trauma trials and observational IPV studies. Clinical sites that will enroll patients for the PRAISE-2 pilot study include the Hamilton General Hospital in Hamilton Ontario, St. Michael’s Hospital in Toronto Ontario, Foothills Medical Centre in Calgary Alberta, Deventer Hospital in Deventer the Netherlands, University Hospital Vall d’Hebron in Barcelona Spain, and Hospital District of Helsinki in Helsinki Finland. All recruiting sites have worked with the Methods Centre previously on at least one large-scale multinational trauma trial and/or IPV study.

### Protecting against sources of bias

#### Ensuring protocol adherence

Prior to starting the study, site investigators and study personnel will attend an investigators meeting, either in-person or by teleconference, to review the study protocol and discuss enrollment and adherence strategies. The Methods Centre will send out regular study newsletters to each clinical site updating them on the overall study progress, summarizing their clinical site’s progress, and thanking them for their continued support. Study personnel will keep daily records of all patients that were eligible but not enrolled in the study (missed) and the reason why. Study personnel will record this information on the case report forms and submit them to the McMaster University-based Methods Centre on a regular basis. Methods Centre personnel will contact any centres with high rates of missed patients to discuss procedures and to establish solutions to any problems. Sites will receive regular quality control reports from the Methods Centre.

#### Ensuring data quality

All study personnel will participate in a training session prior to study commencement to ensure consistency in study procedures, including data collection and reporting. Site investigators can contact the Methods Centre at any time to resolve any problems or questions that arise. We will use an electronic data collection system with quality and logic checks, supplemented with regular manual data quality checks. Study personnel at the Methods Centre will review data for completeness and quality from clinical sites daily. The Methods Centre personnel will follow-up with quality control reports on a regular basis.

#### Maximizing patient follow-up

**Fig. 2:**
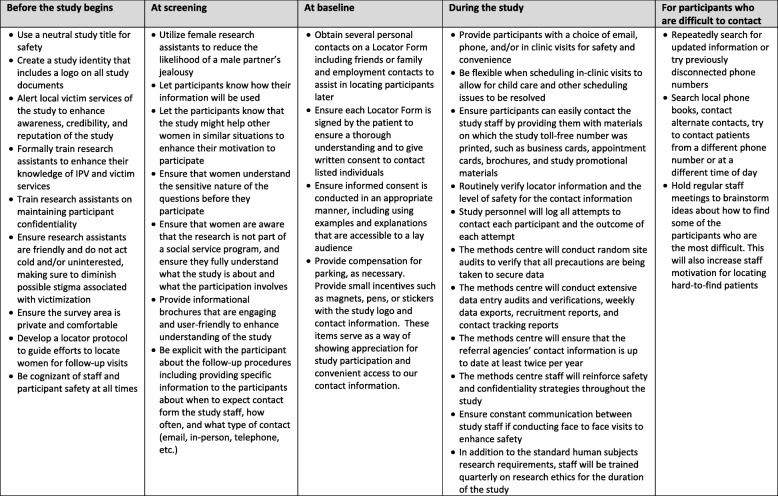
PRAISE-2 enrolment and follow-up enhancement strategies

As previous studies have reported high loss to follow-up rates with IPV victims [35], we will implement a strategy designed to minimize loss to follow-up adapted from Logan et al. [36] (Fig. 2) to reduce bias associated with loss to follow-up. Logan et al. were able to achieve nearly a 75% recruitment rate and a 94% follow-up rate after 1 year in a population of severely abused women [36]. We have previously used the majority of these strategies to maximize follow-up in multicentre studies [37]. Main features of this strategy include (1) excluding individuals who are very likely to present problems with follow-up; (2) prior to leaving the surgical clinic, as well as their own telephone number, each patient will provide the name and address of alternate contacts who are likely to be aware of the patient’s whereabouts; (3) patients will receive a reminder card for their next follow-up visit from the clinical research coordinator; and (4) follow-up visits will coincide with standard fracture clinic visits. Alternatively, patients can complete the major study questionnaires over the phone.

#### Identifying IPV status

A bias toward under-reporting IPV is possible with the self-reporting nature of identifying IPV; however, we were able to elicit a disclosure of abuse in the past year from one in six women in the original PRAISE study which is similar to estimates of IPV in other specialties. Our previous studies show that our method of identifying IPV victims is more sensitive than other common methods of identifying IPV [28]. Because of this, we are confident in our ability to correctly identify the majority of IPV victims. Considerations with respect to confidentiality will be addressed during data collection to reduce bias when participants are completing the questionnaire. Participants will be approached by a female research coordinator, and the consent process and the completion of the questionnaires will take place alone in a private location to reduce influence from others. A female research coordinator is used to make the participant feel safer and more comfortable with disclosure. In our POSITIVE study [38], we found that 82% of female fracture clinic patients would prefer to speak to a female about IPV.

#### Independent blinded adjudication

An independent adjudicator blinded to patients’ IPV status will review radiographs and clinic notes to confirm injury-related complications where necessary.

### Statistical plan

#### Sample size determination

The sample size for the pilot study is primarily based on feasibility considerations. Based on data from the original PRAISE study, 1 in 3 women presenting to fracture clinics have a lifetime history of IPV, 1 in 6 have a history of IPV in the past year, and 1 in 50 women present to fracture clinics because of an IPV-related injury [14]. Based on the statistic that 1 in 50 women present to fracture clinics because of an IPV-related injury, we aim to recruit 50 women at each of 5 sites (250 total). We determined a priori that the study will be feasible if loss to follow-up is less than 15% and adherence to study windows is 75% or greater. We believe that our loss to follow-up will be about 10%; therefore, using the confidence interval approach suggested by Thabane et al. [39], we require 214 patients to achieve a 5% margin of error (which will generate a confidence interval that excludes 15%). We believe that the adherence to study windows will be over 80%; therefore, we require 214 patients to achieve a 6% margin of error (which will generate a confidence interval that excludes 75%). Therefore, 250 participants will be sufficient to assess our feasibility outcomes. We intend to use data from the pilot study to inform the definitive sample size calculation.

#### Primary analysis

A summary of objectives, outcomes, and analyses is presented in Table 3. We will report feasibility outcomes descriptively by site as proportions with 95% CIs. We will also report whether each feasibility outcome met the criteria for feasibility success.

**Table 3:** Summary of objectives, outcomes, success criteria, and analyses

Primary			
Objective	Outcome	Success criteria	Analysis
Feasibility	Recruitment	Each site should recruit 50 participants in 12 months or less	Descriptive—proportions with 95% CI
	Adherence to visit windows	At least 75% of study visits should be within the defined windows	
	Participant retention	Loss to follow-up should remain under 15%	
	Data completeness	Questionnaire completion rates should remain over 80%	
Secondary			
Objective	Outcome	Analysis	
Determine how a history of IPV affects injury-related complications	Injury-related complications	Proportions of patients experiencing injury-related complications by group with 95% CI; logistic regression.	
Determine how a history of IPV affects return to pre-injury function	Return to pre-injury function	Proportion of patients achieving return to pre-injury level of function in each group at baseline, 3 months, 6 months, and 12 months with 95% CI; logistic regression.	
Determine incident cases of IPV	New IPV disclosures	Incidence statistic with 95% CI.	
Determine how a history of IPV affects health care and support service use	Utilization and associated costs of support services and hospitalizations	Proportions of women using each service per group over 12 months, median number of times that participants used each service with IQR. Estimated mean costs with 95% CI in each group and bootstrap differences between those with a history of IPV and those without with 95% CI.	
Determine how patterns of IPV change over time	Changes in abuse severity/frequency and type	Proportion of patients who experienced no abuse, a stable level of abuse, escalating abuse, and de-escalating abuse over 12 months with 95% CI; graphically.	
Determine how a history of IPV affects HRQL after a musculoskeletal injury	EuroQuol-5 Dimensions (EQ-5D)	Mean change in HRQL from baseline to the 3 month, 6 month, and 12 month visits, by group with 95% CI.	
Determine how abused women’s stage of change changes over time	Domestic Violence Survivor Assessment (DVSA)	Change in stage of change from baseline at 3 months, 6 months, 9 months, and 12 months.	

*CI* confidence interval, *IQR* interquartile range, *HRQL* health-related quality of life

#### Secondary analyses

The analyses of clinical outcomes will be exploratory in nature since the primary focus is on assessing feasibility. We will therefore not present *p* values for comparative analyses. We will not carry out any imputation for missing data in this pilot study.

- Injury-related complications—We will present proportions of patients experiencing injury-related complications in each group (i.e. women with and without a history of IPV) over 12 months with 95% CI. We will also perform an exploratory logistic regression analysis.
- Return to pre-injury function—We will report the proportion of patients achieving return to pre-injury level of function in each group at baseline, 3 , 6 , and 12 months with 95% CI. We will also perform an exploratory logistic regression analysis.
- New IPV disclosure*—*New IPV disclosures will be reported as an incidence statistic with 95% CI.
- Utilization and costs of health, legal, and social support services—Will be reported descriptively as proportions of women using each service per group over 12 months as well as the median number of times that participants used each service with interquartile range (IQR). Direct costs will be derived by assigning costs to adjudicated injury-related complications and self-reported utilization of health care services based on provincial case costing registries and health care provider schedule of benefits. All remaining direct costs will be estimated by multiplying self-reported units of utilizations (e.g. visits to social worker, use of mental health services) by an estimate of the cost per unit of service based on provincial or national average charges. Indirect costs will be calculated using self-reported annual income and return to function. Costs will be presented as means with 95% CIs, and histograms. Due to the non-normality of cost data, non-parametric bootstrap estimates will be used to present the difference in mean costs between those with and without a history of IPV. Multivariable sensitivity analysis will be conducted by using 95% CI and reported cost ranges for input parameters. All costs will be inflated to 2018 Canadian dollars using the appropriate price indices.
- Changes in IPV type and severity—We will report the proportion of patients who experienced no abuse over 12 months, a stable level of abuse over 12 months, escalating abuse, and de-escalating abuse over 12 months with 95% CI. We will present this graphically.
- Health-related quality of life—We will report the mean change in HRQL from baseline to the 3-, 6-, and 12-month visits by group with 95% CI. We will also estimate utility, which will be modelled over the course of 1-year follow-up, using 3-, 6-, and 12-month EQ-5D scores and standard trapezoidal rules. Utility will be presented as quality adjusted life years (QALYs) for each group, with 95% CI, where 1 represents full health and 0 represents death. The difference between each group will be presented as QALYs lost.
- Stages of change—We will report the change in stage of change from baseline at 3, 6, 9, and 12 months for patients who report that their current relationship is or was abusive.

### Ethical considerations

#### Informed consent and ethics approval

We have secured approval from the Hamilton Integrated Research Ethics Board (project # 15–383) for the Methods Centre and each participating clinical site will obtain approval from their local ethics board before initiating this study. Each participant will sign an informed consent form (ICF) before participating in the study according to local ethics protocols and the ICF will be worded in lay terms. To maximize the opportunity for free and informed consent while respecting privacy and confidentiality, the informed consent process will only take place privately. Potential participants will not be invited to join the study if the clinical research coordinator is not able to secure an opportunity when the individual is alone long enough to adequately explain the study and obtain informed consent. By approaching the potential participant in private, the participant also has the opportunity to provide free consent in the absence of significant others that may affect her decision to participate.

#### Privacy and confidentiality

At every step of the PRAISE-2 study, privacy, and confidentially will be paramount. Due to the sensitive nature of the research topic, we will be certain to exercise caution when recruiting individuals to participate in the study. Women are often fearful of disclosing that they are a victim of IPV for fear of retaliation from the offender, stigmatization by the individuals that she discloses to, embarrassment, and police involvement [40]. For safety reasons, women will only be allowed to participate in the study if they are able to complete the questionnaires in a private location. Research coordinators will not mention words “abuse” or “violence” at any point unless they are in a private location. This approach has been successfully used in other IPV studies [14, 15, 41]. Paper CRFs will be stored in a secure location at each clinical site and will be destroyed per local regulations after the study’s completion. Privacy and confidentiality will further be secured by assuring that research numbers will be used in place of personal identifiers when clinical sites communicate with the Methods Centre.

#### IPV disclosure

For ethical reasons, if a woman discloses that she has experienced IPV and wishes to speak to her surgeon about it, research personnel will notify the attending surgeon (with the participant’s permission) and the surgeon will offer support if needed using his/her clinical judgment. The research coordinator at the Methods Centre, who has over 7 years of experience coordinating IPV studies, and an IPV expert, will conduct training calls or in-person training sessions with surgeons and research personnel before the study begins and over the course of the study so they are able to effectively respond to IPV disclosures. Surgeons will be provided with training slides that they can refer back to if needed, as well as a set of instructions on how best to assist IPV victims if they require assistance to be posted in the surgical clinic surgeon area. Information provided to surgeons will include contact information for a community-based and a hospital-based social worker, and tips on what to say to women who disclose IPV. This set of instructions was developed in partnership with a community social worker and a hospital-based social worker and has been used in previous and ongoing IPV research.

## Discussion

Previous studies have assessed physical health outcomes of IPV victims, but they are limited by their cross-sectional design and by only assessing general physical health outcomes. Based on the international attention earned by the original PRAISE study [14], we believe that orthopaedic surgeons and others who treat injured women are interested in gaining insight into how IPV affects their patients’ injury-related outcomes. This pilot study will begin to fill some of the gaps in the literature faced by health care professionals who treat injured women who have experienced IPV. It will also provide much-needed feasibility data for recruiting, following, and measuring outcomes in a longitudinal IPV study of injured women in a fracture clinic setting.

We believe a pilot study is a critical step to ensure feasibility of future longitudinal IPV research among injured women. In previous studies of IPV victims, patient follow-up has typically been difficult, with one large otherwise high-quality trial losing almost 40% of their study sample over 18 months. We propose a plan to minimize loss to follow-up (Fig. 2) based on the strategies developed by Logan et al. [36] and adapted by Madden et al. [37], and we will take this opportunity to test and refine this strategy. We will use the information gained in the pilot study to determine the feasibility of a larger definitive study and to refine recruitment, follow-up, measurement, and data collection strategies that will be useful in not only prospective cohort studies in this population, but also future planned interventional research in our IPV research program. The ultimate goal of our research program in IPV is to reduce further violence and injuries. We aim to accomplish this by evaluating an identification and support program for victims of IPV in fracture clinics. Health care professionals, researchers, and health policy makers require high-quality, evidence-based information to guide their decisions. The current pilot prospective cohort will begin to fill some of these gaps in knowledge. In order for a screening and support program to be successful, health care professionals need to understand whether having a musculoskeletal injury can lead to new or worsening IPV, how patterns and types of IPV can change over time, and the types of services victims utilize, have access to, and need.

A key limitation of this study is the reliance on self-reporting of IPV status. We have attempted to limit bias by centrally adjudicating fracture-related adverse events by an independent blinded orthopaedic surgeon; however, IPV status cannot be centrally adjudicated. Despite this limitation, we are confident that women are comfortable answering the three direct questions that we propose to use, based on previous studies in a similar population.

## Conclusion

The PRAISE-2 pilot study is the first step toward determining how experiences of IPV affect orthopaedic injury outcomes such as injury-related complications. This study will inform future observational and interventional longitudinal studies on IPV in a fracture clinic setting that will engage physicians, surgeons, nurses, social workers, physiotherapists, and chiropractors from around the world to increase awareness of how IPV affects their patients’ injury outcomes.
